# Depressive symptoms in non-alcoholic fatty liver disease are identified by perturbed lipid and lipoprotein metabolism

**DOI:** 10.1371/journal.pone.0261555

**Published:** 2022-01-06

**Authors:** Daniel E. Radford-Smith, Preya J. Patel, Katharine M. Irvine, Anthony Russell, Dan Siskind, Daniel C. Anthony, Elizabeth E. Powell, Fay Probert

**Affiliations:** 1 Department of Pharmacology, University of Oxford, Oxford, United Kingdom; 2 Department of Chemistry, University of Oxford, Oxford, United Kingdom; 3 Department of Psychiatry, Warneford Hospital, University of Oxford, Oxford, United Kingdom; 4 Institute for Liver and Digestive Health, University College London, London, United Kingdom; 5 The Liver Unit, Newcastle Upon Tyne Hospitals NHS Foundation Trust, Newcastle upon Tyne, United Kingdom; 6 Centre for Liver Disease Research, The University of Queensland, Brisbane, Australia; 7 Mater Research, The University of Queensland, Brisbane, Australia; 8 Department of Diabetes and Endocrinology, Princess Alexandra Hospital, Brisbane, Australia; 9 Centre for Health Services Research, The University of Queensland, Brisbane, Australia; 10 School of Clinical Medicine, The University of Queensland, Woolloongabba, QLD, Australia; 11 Metro South Addiction and Mental Health Service, Woolloongabba, QLD, Australia; 12 Department of Gastroenterology and Hepatology, Princess Alexandra Hospital, Brisbane, Australia; CIC bioGUNE, SPAIN

## Abstract

Non-alcoholic fatty liver disease (NAFLD) and depression are common disorders and have bidirectional contributing relationships to metabolic syndrome. We aimed to determine whether a fasting serum signature of recent, self-reported depressive symptoms could be identified in a heterogeneous NAFLD cohort using nuclear magnetic resonance (NMR)-based metabolomics integrated with clinical chemistry. Serum nuclear magnetic resonance (NMR) metabolite profiles and corresponding clinical chemistry were compared between patients with depressive symptoms in the last 12-months (n = 81) and patients without recent depressive symptoms (n = 137 controls) using multivariate statistics. Orthogonal partial least squares discriminant analysis (OPLS-DA) of the biochemical and metabolomic data identified NAFLD patients with recent depression with a cross-validated accuracy of 61.5%, independent of age, sex, medication, and other comorbidities. This led to the development of a diagnostic algorithm with AUC 0.83 for future testing in larger clinical cohorts. Serum triglycerides, VLDL cholesterol, and the inflammatory biomarker GlycA were key metabolites increased in patients with recent depressive symptoms, while serum glutamine level was reduced. Here, serum NMR metabolite analysis provides a link between disturbed lipid metabolism, inflammation, and active mental health issues in NAFLD, irrespective of disease severity.

## Introduction

With an estimated worldwide prevalence of one in four individuals, non-alcoholic fatty liver disease (NAFLD) is the most common chronic liver disorder [1] and has a close, bidirectional association with other obesity-related metabolic disorders [2]. Depression, similarly to NAFLD, is a common and complex disorder, a global health and economic burden, and can substantially reduce the quality of life of those afflicted [3]. The co-occurrence of major depressive disorder (MDD) with obesity [4] and metabolic syndrome [5] is common and well documented by both longitudinal and cross-sectional studies. This association is suggestive of an interactive relationship with overlapping pathophysiological mechanisms related to metabolic dysfunction and chronic, low-grade “metabolic” inflammation [4]. Emerging evidence points to a bidirectional association between NAFLD and MDD; NAFLD has been found to increase the risk of anxiety and depression independently of other comorbid diseases [6], and depressed individuals are more likely to have NAFLD than non-depressed individuals independent of diabetes and obesity [7].

In this study, we performed serum nuclear magnetic resonance (NMR)-based metabolomics on 218 NAFLD patients. NMR allows for the measurement of lipoprotein particle concentration, as opposed to lipoprotein cholesterol content. Many other low-molecular-weight metabolites are also measured simultaneously that are beyond the resolution of standard serological lipid and biomarker panels, including factors involved in immunological and neurological pathways. We aimed to determine whether people with NAFLD and recent (<12 months) depressive symptoms could be distinguished from those without recent depressive symptoms based on clinical or metabolic biomarkers, with a view to gaining further insight into the links between depression and chronic metabolic dysfunction. Here, we report that NAFLD patients with or without recent depressive symptoms can be stratified based on differences in serum metabolite levels, irrespective of disease severity. This suggests that the depression associated with NAFLD is not simply a downstream consequence of NAFLD-induced alterations to the metabolite pool, but a discrete condition with additive metabolic sequelae.

## Subjects and methods

### Subjects

The study cohort comprised patients with NAFLD recruited from type 2 diabetes (T2DM) clinics and at-risk populations in primary care between October 2015 and August 2017, who had a fasting serum sample without freeze-thaw available for analysis (n = 218). The clinical characteristics of the source population have been previously described [8]. Informed written consent was obtained from each patient and the protocol was approved by the Metro South Health and The University of Queensland Human Research Ethics Committees (HREC/15/QPAH/301; UQ2015001047).

### Clinical assessment and self-reported depression

Clinical assessment included anthropometric measurements; routine hematologic, biochemical, and serologic tests; and liver ultrasound. Transient elastography was performed using FibroScan technology (Echosens, Paris, France) as previously described [8]. Medical history was obtained during the initial consultation in the liver clinic using a structured questionnaire (S1 Appendix). Question items included self-reported sociodemographic characteristics, use of medications, previously diagnosed liver disease, and medical conditions including a clinical diagnosis of depression and whether they had depressive symptoms in the previous 12 months.

### Nuclear magnetic resonance (NMR) spectroscopy

Blood was collected on the morning of clinical assessment into BD Vacutainer SST tubes (BD 368968, NJ, USA), allowed to clot at room temperature for 3–4 hours, and centrifuged at 1,300xg for 10 minutes at room temperature. The supernatant (serum) was immediately frozen on dry ice. For the metabolomic analysis, samples were transported from Australia to the United Kingdom on dry ice. In the intervening periods between sample collection, shipment, and the day of analysis, samples were stored at -80°C. Serum samples were thawed at room temperature and mixed by pipetting, before 150uL of serum was added to 450uL of 75 mM sodium phosphate buffer prepared in D_2_O (pH 7.4). Samples were then transferred to a 5mm borosilicate glass NMR tube (Norell 502–7). All NMR samples were prepared on the day of analysis by the same individual. The time between thaw and NMR data acquisition did not exceed 9 hours. All NMR experiments were acquired using a 700-MHz Bruker AVIII spectrometer equipped with a ^1^H [^13^C/^15^N] TCI cryoprobe (Department of Chemistry, University of Oxford) under parameters described previously [9].

### Statistical analysis

Significance level was set at *p*<0.05. Multivariate OPLS-DA was performed on clinical and metabolome data using in-house R scripts and the *ropls* package [10]. Models were computed with matched class sizes and validated on independent test sets using a 10-fold external cross-validation strategy with 100 repetitions (S1 Fig), as previously described [11]. The accuracies of OPLS-DA models were compared to a null distribution (by randomising sample/group assignments) using the two-sided Kolmogorov-Smirnov test. Clinical chemistry data were retained in the multivariate analysis of serum metabolites to compare the variable importance in projection (VIP) scores between clinical and metabolomic data.

Individual biochemical parameters were analysed using one-way analysis of variance with Tukey’s multiple comparisons test, or the Chi-squared test. Univariate analysis of key serum metabolites was performed between those with (n = 81) or without (n = 137) recent depressive symptoms using Welch’s unequal variances *t*-test and Bonferroni’s method of correction for multiple testing. Outliers were detected using the ROUT method (Q = 0.1%) and removed from post-hoc univariate analyses. Receiver operating characteristic (ROC) curves were built using the pROC package in R [12] with 95% confidence interval (CI). When comparing recent depression and no/lifetime depression, the optimal threshold for model accuracy based on all data was determined using the Youden index.

## Results

### Clinical characteristics including liver stiffness measurement do not identify recent or lifetime depressive symptoms in patients with NAFLD

Of the 218 patients with NAFLD, 81 patients had recent self-reported depressive symptoms within the last 12-months at the time of blood sampling. 30 patients had a history of depression (without recent depressive symptoms), and 107 patients had no history of depression.

Patients with NAFLD and recent depressive symptoms were younger (p = 0.0007 compared to lifetime depression; p = 0.012 compared to no depression), with a significantly higher fasting serum triglyceride level (p = 0.022 compared to lifetime depression; p = 0.0015 compared to no depression) and trend toward lower fasting serum high-density lipoprotein (HDL; p = 0.099 compared to no depression). As expected, there was a difference in antidepressant use between those with and without a history of depression (p<0.0001). However, there were no significant differences in current use of medications for dyslipidaemia, presence of metabolic comorbidities, or liver disease severity (Table 1).

**Table 1:** Demographic and clinical characteristics of the patient cohort according to self-reported depressive symptoms.

	Depressive symptoms			
	Recent depressive symptoms (<12months) N = 81	Lifetime depression (no symptoms for >12months) N = 30	No Depression N = 107	p-value
**Sex (% female) ^‡^**	46.9%	63.3%	38.3%	0.047*
**Age (yrs)^†^**	55 (45–61)	65 (55.75–71.25)	61 (51.8–70)	0.0004***
**BMI (kg/m^2^)^†^**	33 (29.3–38.0)	30.3 (28.2–37.8)	33.0 (29.1–38.7)	0.22
**eGFR (mL/min/1.73m^2^)^†^**	90 (80–90)	87 (70–90)	88 (66–90)	0.10
**Serum ALT (IU/mL)^†^**	31 (22–51)	27 (19–39)	28 (20–47)	0.53
**Serum AST (IU/mL)^†^**	21 (15–28)	21 (16–29)	21 (15–32)	0.85
**HBA1c (%)^†^**	7.7 (6.6–9.0)	6.9 (6.1–8.4)	7.2 (5.9–8.8)	0.25
**Serum Ferritin (μg/L)^†^**	70 (38–141)	58 (25–146)	91 (48–159)	0.27
**Serum Cholesterol (mmol/L)^†^**	4.5 (3.6–5.5)	4.6 (3.5–5.8)	4.3 (3.5–5.1)	0.23
**Serum Triglycerides (mmol/L)^†^**	2.0 (1.6–2.9)	1.7 (1.2–2.0)	1.6 (1.0–2.3)	0.0009***
**Serum HDL (mmol/L)^†^**	1.0 (0.8–1.1)	1.2 (0.9–1.3)	1.1 (0.9–1.3)	0.086
**Serum LDL (mmol/L)^†^**	2.6 (1.7–3.2)	2.5 (1.8–3.8)	2.2 (1.7–3.0)	0.41
**LSM kPa^†^^§^**	5.6 (4.8–7.9)	4.8 (4.4–5.7)	6.1 (4.7–8.6)	0.10
**T2DM n (%)^‡^**	72 (88.9)	23 (76.7)	86 (80.4)	0.19
**Dyslipidaemia n (%)^‡^**	77 (95.1)	29 (96.7)	102 (95.3)	0.94
**Metabolic Syndrome n (%)^‡^**	67 (82.7)	25 (83.3)	88 (82.2)	0.99
**Current antidepressant use n (%)^‡^**	37 (45.7)	12 (40.0)	0 (0.0)	<0.0001****
**Current statin use n (%)^‡^**	51 (63.0)	17 (56.7)	64 (59.8)	0.81
**Current fibrate use n (%)^‡^**	12 (14.8)	2 (6.7)	10 (9.3)	0.35

^†^Continuous data (median [IQR]) analysed using one-way analysis of variance

^‡^Categorial data analysed using Pearson’s chi-squared test.

^§^LSM (liver stiffness measurement) presented for 197/218 patients with a reliable measurement.

BMI, body mass index;eGFR, estimated glomerular filtration rate; ALT, alanine aminotransferase; AST, aspartate aminotransferase; HBA1c, glycated haemoglobin; HDL, high-density lipoprotein; LDL, low-density lipoprotein; T2DM, type 2 diabetes mellitus.

While OPLS-DA of all clinical chemistry variables identified a significant difference between NAFLD patients with and without recent depression (p<0.0001; S2A and S2B Fig) the multivariate model did not afford more information than the univariate analysis in this case. Average VIP values determined from the ensemble of OPLS-DA models are shown in S1 Table. Indeed, inspection of the individual biochemical parameters classifying recent depressive symptoms (n = 81) from no recent depressive symptoms (n = 137; S2 Table) revealed a predominant role of serum metabolic factors including triglyceride level (area under curve [AUC] 0.64 [95%CI 0.57–0.71]) and HDL (AUC 0.61 [95%CI 0.53–0.68]). Alternatively, when patients with a lifetime history of depression were combined with those with recent depression (combined n = 111) and compared to those with no history of depression (n = 107), the mean OPLS-DA accuracy did not perform significantly better than random chance (p = 0.20; S2C and S2D Fig).

### Incorporating serum metabolomic data with clinical chemistry enables identification of recent depressive symptoms primarily through lipoprotein resonances

**Fig 1:**
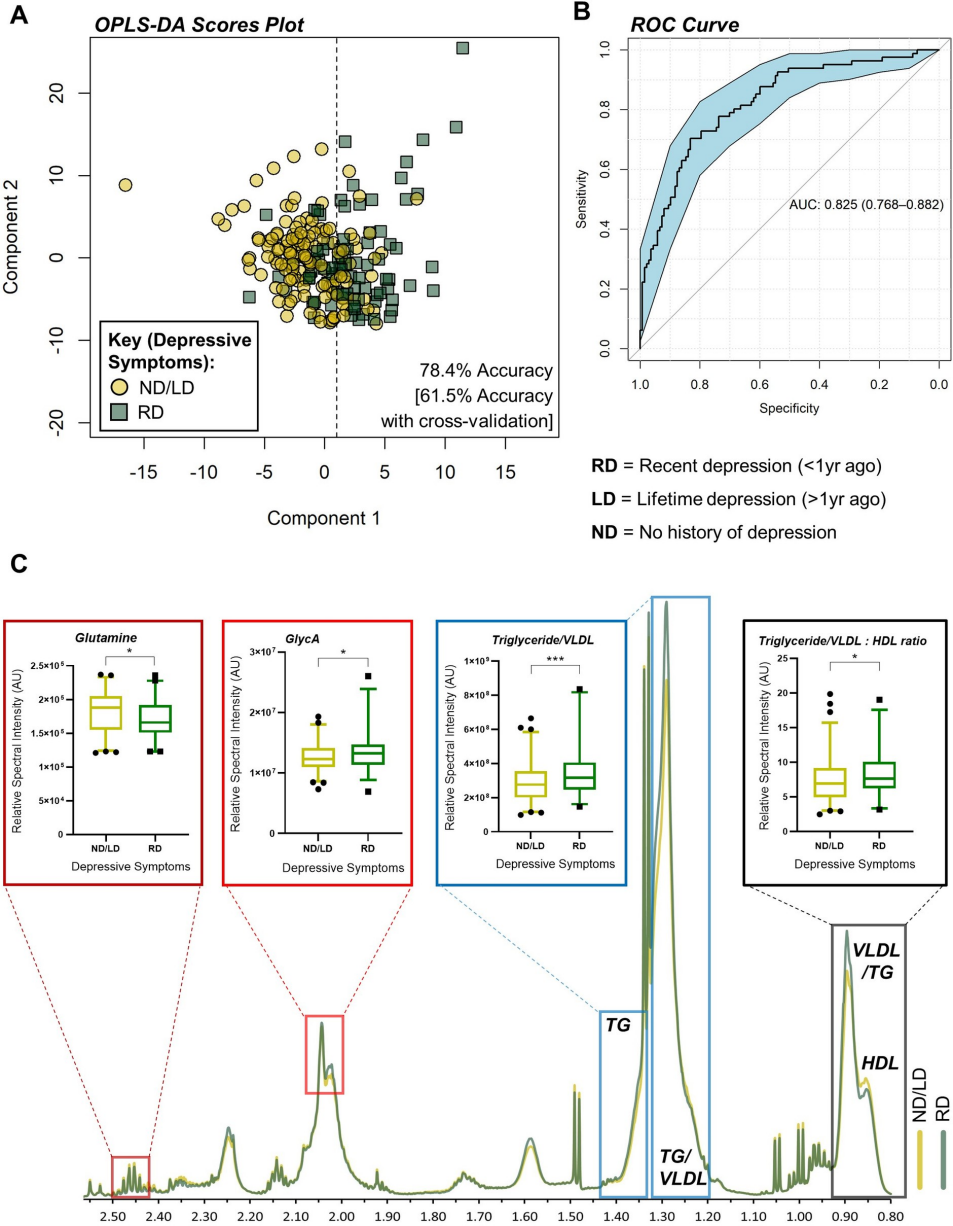
Nuclear magnetic resonance spectroscopy (NMR) identifies a serum metabolic signature of recent depressive symptoms in NAFLD. **A)** Orthogonal partial least squares discriminate analysis (OPLS-DA) scores plot, based on integrated clinical and metabolomic data, coloured by depressive symptoms. R^2^X = 79.0±1.8%, R^2^Y = 27.4±2.9%, Q^2^ = 4.4±5.3%. **B)** Receiver operating characteristic (ROC) curve for the OPLS-DA scores, classifying recent vs. lifetime/no depressive symptoms. **C)** Visualisation of NMR spectrum illustrating the key resonance peaks contributing to the OPLS-DA model, including serum glutamine (Welch’s unequal variances *t*-test with Bonferroni’s correction, p = 0.048), VLDL/triglycerides (p = 0.0084), the VLDL/triglyceride:HDL ratio (p = 0.034) and GlycA (p = 0.013). ROC curve shows AUC ± 95% confidence intervals. N = 81 (recent depression) and 137 (lifetime/no depression).

Multivariate analysis (OPLS-DA) of serum metabolites, simultaneously quantified by ^1^H-NMR spectroscopy, and clinical chemistry was used to generate models distinguishing recent self-reported depressive symptoms from all other NAFLD patients with no recent depressive symptoms (Fig 1A). Permutation testing verified that this model performed better than chance (mean cross-validated accuracy 61.5% [95%CI 60.9–62.1], p<0.0001) and 10-fold external cross-validation confirmed that the model performance was not a result of over-fitting the data (S3A Fig). Average VIP values determined from the ensemble of OPLS-DA models are shown in S3 Table. The key serum metabolites identified from the OPLS-DA with cross-validation are shown in Table 2. As this integrated metabolomic and clinical chemistry model performed best in classifying NAFLD patients with and without recent depressive symptoms, we performed an OPLS-DA of all the data to produce a single model that could be applied to future cohorts. From this analysis, a ROC was used to identify the optimal threshold based on the Youden index along the x-axis of the scores plot, with an accuracy of 78.4% (Fig 1A) and an AUC value of 0.83 (95%CI 0.77–0.88, Fig 1B). NAFLD patients without recent depressive symptoms included those with and without a lifetime history of depression. These patients showed equal dispersion on the left side of the scores plot (S3B Fig), suggesting that lifetime depression is not as pertinent to the serum metabolome as recent depressive symptoms. Indeed, a weaker association (accuracy 55.9% [95%CI 55.4–56.4], p<0.0001) was identified by OPLS-DA between NAFLD patients with any history of depression (n = 111) and those without (n = 107; S4A and S4B Fig). Average VIP values determined from the ensemble of OPLS-DA models for lifetime depression are shown in S4 Table.

**Table 2:** Summary of discriminatory metabolites.

Metabolite [bin ppm]	VIP score
HDL [0.81–87]	1.77
VLDL/Triglyceride [0.87–0.92]	1.85
Triglyceride/VLDL [1.22–1.32; 1.34–1.43]	1.82; 1.89
GlycA [2.04–2.05]	1.51
Glutamine [2.43–2.47]	1.77

Data showing spectral bin assignment and corresponding VIP score for key serum metabolites. HDL, high-density lipoprotein; VLDL, very low-density lipoprotein; GlycA, glycoprotein acetylation.

Further inspection of the variables most contributing to the OPLS-DA model in Fig 1A revealed a predominate contribution of lipid resonances (S3 Table). Triglyceride/very low-density lipoprotein (VLDL) (-CH_2_-)_n_ resonances were increased in NAFLD patients with recent depressive symptoms relative to those without (Fig 1C). Those with recent depressive symptoms had significantly higher levels of VLDL cholesterol compared to HDL cholesterol, and lower levels of serum glutamine. In addition, glycoprotein acetylation (GlycA), an NMR-based biomarker of peripheral inflammation [13], was significantly increased in patients with recent depressive symptoms, compared to those without (Fig 1C).

Although those with recent depressive symptoms were younger than those without recent depressive symptoms (Table 1), the OPLS-DA metabolomic model correctly identified NAFLD patients without recent depression with high sensitivity independently of age (S5A Fig). To verify the OPLS-DA model controlling for age at sampling, an age and sex-matched subset (n = 70 per group) was randomly selected. The subsequent OPLS-DA model remained highly discriminatory (S5B Fig) with an AUC value of 0.82 (95%CI 0.75–0.89, S5C Fig). Metabolites contributing to this model were representative of the original OPLS-DA model (S5 Table).

Similarly, we verified that the original OPLS-DA model was not affected by sex (S6 Fig) or antidepressant treatment (S7 Fig). Similar proportions of those with recent and non-recent depression were on antidepressants (Table 1), though those with non-recent depression had a serum metabolic profile more akin to those with no history of depression (S3B Fig).

## Discussion

Despite growing evidence for the association between NAFLD and depression over the last decade [6], the presence of depressive symptomology, particularly subclinical, is not typically assessed in the liver clinic. To gain further insight into the links between depression and chronic metabolic dysfunction, we performed serum NMR metabolomics to investigate metabolic perturbations associated with recent depressive symptoms in patients with NAFLD. Here, we report a serum metabolic signature of recent (<12 months) self-reported depressive symptoms, distinct from those without recent depressive symptoms, with or without a lifetime history of depression (cross-validated OPLS-DA accuracy 61.5%).

The key variables driving identification of recent depressive symptoms included multiple triglyceride and lipoprotein resonances, particularly total triglycerides, HDL, and VLDL. A pooled analysis of NMR-based plasma metabolomics from 5,283 patients with depression and 10,145 control subjects identified similar biomarkers [14]: higher levels of VLDL cholesterol, glycerides, diglycerides, triglycerides, total fatty acids, and the inflammatory marker GlycA were associated with depression, as were lower levels of HDL-cholesterol. Interestingly, many of these associations were strengthened when only current depression (3,265 patients) was included, defined as blood sampling within one month of diagnosis by clinical interview or questionnaire. A meta-analysis of depression in people with metabolic syndrome also identified a significant positive association between hypertriglyceridemia and depression, and a negative association between HDL levels and depression [5]. The original studies pertaining to this meta-analysis used a mixture of self-reporting and structured interviews to classify depression. Associations between depression and metabolic perturbations were stronger in self-reported individuals rather than those diagnosed using structured clinical interviews, or by a clinician. We demonstrate that a similar serum metabolic signature of depression is detectable in a patient cohort with pre-existing metabolic dysfunction.

Alterations in the lipid spectrum identified in the present study may represent shared metabolic abnormalities linking depression to cardiometabolic diseases. In rodents, liver fatty acid synthesis may be increased by chronic stress due to changes in transcriptional activity of key metabolic enzymes [15]. Chronic unpredictable mild stress (CUMS) in rodents has been shown to significantly elevate plasma ALT and AST levels, triglycerides, and other lipid constituents compared to controls [16]. Similarly, chronic stress in mice has been found to increase hepatic triglyceride and cholesterol levels whilst decreasing overall visceral fat mass [17]. Increased lobular inflammation, hepatocyte injury, and serum pro-inflammatory cytokine levels were also noted in the stressed animals compared to controls. These preclinical studies reinforce the bidirectional association between NAFLD, cardiometabolic disease risk, and depression, which may be driven by alterations to peripheral inflammation and lipid metabolism.

Moreover, we found GlycA levels to be significantly increased in NAFLD patients with current depression. This is consistent with other reports of an association between GlycA and depression symptom severity as well as depression persistence [18, 19], and likely relates to the relationship between metabolic dysfunction, dyslipidaemia, depression, and the chronic, low-grade inflammation intrinsic to these conditions [20]. Lastly, we found serum glutamine to be reduced in people with NAFLD and current depression, compared to patients with NAFLD without current depression. Reduced serum glutamine has been identified as a metabolic biomarker for major depression [21] and may be correlated with brain gamma aminobutyric acid (GABA) levels [22]. Another study between depressed and non-depressed individuals found no difference in serum glutamine level, although the sample size was considerably smaller [23]. We identify these substrates as potential targets for the prevention and treatment of depression as well as the cardiometabolic comorbidities associated with this chronic disorder.

Few studies have investigated clinicopathological and metabolic features of NAFLD patients with and without depression. In 2013, Youssef and colleagues [24] identified subclinical depression in over half of 567 patients with NAFLD, and clinical depression in 14%. Both subclinical and clinical depression were associated with more severe hepatocyte ballooning (reflecting hepatocellular injury) in liver biopsy sections. Tomeno and colleagues in 2014 identified a higher grade of steatosis, though no differences in hepatocellular ballooning or fibrosis were found in NAFLD patients with depression (n = 32) compared to without (n = 226) [25]. Additionally, individuals with stable depression (defined as full or partial remission) showed a greater improvement in alanine aminotransferase and gamma-glutamyltransferase levels after a year of standard care than patients with unstable depression (not in full or partial remission) [25]. This may indicate that NAFLD patients comorbid with depression do not respond to medical intervention as well as NAFLD patients without an affective disorder, particularly in those with recent rather than a lifetime history of depressive symptoms.

Whilst depression has not been found to increase the likelihood of advanced fibrosis [7], managing comorbid depression remains an important facet of clinical care. Individuals with depression are more likely to lead an unhealthy lifestyle and less likely to adhere to a medical regimen, particularly those involving lifestyle adjustments [26]. In patients with cardiovascular disease, the leading cause of premature death in NAFLD patients [2], persistent depressive symptoms (as opposed to none or remitting) predicted worse health-related quality of life after one year [27]. Therefore, identifying and treating NAFLD patients with recent depressive symptoms may improve quality of life relating to comorbid cardiovascular disease.

A key limitation of this study relates to the reporting of depression. Participants self-reported whether they had received a clinical diagnosis of depression, and whether they had symptoms in the previous 12 months. We used this data to stratify participants into groups with recent, lifetime, or no depressive symptoms. We did not have access to data from a validated depression scale or diagnostic interview, which may limit the diagnostic rigour of this study. However, single item self-report of depression has previously been shown to have reasonable correlation with validated interview schedules for depression [28]. Here, all patient interviews were performed by the same person using the same template, providing consistency. An advantage of our self-report measure is that it may more accurately reflect clinical practice, improving the clinical translatability of our findings to real world NAFLD clinics and sensitively capturing a metabolic signature of depressive symptomology in NAFLD, as was described previously in metabolic syndrome [5].

An additional limitation is the lack of detailed information regarding the dietary intake and physical activity of this NAFLD cohort, leading to uncertainty over the cause-and-effect relationships between depression, NAFLD, and lifestyle habits. Post-prandial inflammation can occur up to twelve hours after eating [29]. Despite highly variable lipid metabolism between individuals, post-prandial serum triglyceride metabolism has been shown to correlate strongly with GlycA levels within individuals, highlighting the important relationship between diet and inflammation [30]. Here, we were unable to determine whether pre-existing depression is driving eating behaviour and metabolic signals, or whether a poorer quality diet has led to inflammation, metabolic dysfunction, and subsequent depression.

Given that lifestyle interventions remain the key tool to halt or reverse liver disease progression in NAFLD, and that treating depression facilitates exercise and weight loss [31], it is important to identify and treat depression in liver clinics. Despite high rates of obesity and metabolic syndrome within the cohort, we identified a robust signature of recent depressive symptoms through altered serum lipid/lipoprotein composition in patients at varying stages of NAFLD. This result is concordant with previous, larger cohorts discriminating depressed from non-depressed individuals, and provides support for a link between disturbed lipid metabolism, liver disease, and active mental health issues. Further work in this area is encouraged to improve understanding of the aetiology of depressive symptoms in patients with metabolic dysfunction, with the aim of identifying novel therapies tailored to this patient population.

## Supporting information

S1 Appendix NAFLD structured questionnaire. (DOC)

S1 Table Mean variable importance in projection scores for recent depression vs. lifetime/no history depression (clinical data only). (DOCX)

S2 Table Relationship between patient clinical and biochemical parameters and recent self-reported depressive symptoms. (DOCX)

S3 Table Mean variable importance in projection scores for recent depression vs. lifetime/no history depression (clinical and metabolomic variables). (DOCX)

S4 Table Mean variable importance in projection scores for lifetime depression (including recent depression) vs. no depression (clinical and metabolomic variables). (DOCX)

S5 Table Mean variable importance in projection scores for recent depression vs. lifetime/no history depression in age-matched subset (clinical and metabolomic variables). (DOCX)

S6 Table Key serum metabolites distinguishing recent depression vs. lifetime/no history depression do not correlate with markers of fibrosis. (DOCX)

S1 Fig Orthogonal partial least squares discriminant analysis (OPLS-DA) methodology flowchart. (TIF)

S2 Fig Orthogonal partial least squares discriminant analysis (OPLS-DA) of clinical chemistry identifies recent, but not lifetime depressive symptoms in this cohort with non-alcoholic fatty liver disease. **A)** OPLS-DA scores plot comparing recent depressive symptoms (in the last 12 months, N = 81) against no recent depressive symptoms in people with or without lifetime depression (N = 137). **B)** Corresponding mean accuracy of OPLS-DA models compared to null distribution, 52.7% [95%CI 52.0–53.4], Kolmogorov-Smirnov test p<0.0001. **C)** OPLS-DA scores plot comparing lifetime/recent depression (N = 111) against NAFLD patients with no history of depression (N = 107). **D)** Corresponding mean accuracy of OPLS-DA models compared to null distribution, 50.3% [95%CI 49.8–50.7], Kolmogorov-Smirnov test p = 0.20.
(TIF)

S3 Fig Orthogonal partial least squares discriminant analysis (OPLS-DA) comparing NAFLD patients with and without recent depressive symptoms. **A)** Mean accuracy of OPLS-DA models (61.5% [CI 60.9–62.1]). 10-fold cross validation and permutation testing with 100 repetitions verified that the model was significantly better than random chance (Kolmogorov-Smirnov test, p < 0.0001). N = 81 NAFLD patients with recent depression, N = 137 patients with no/lifetime depression. **B)** OPLS-DA scores plot coloured by recent (N = 81), lifetime (N = 30), and no history of depression (N = 107) illustrates that the serum metabolic and clinical profile of lifetime depression in non-alcoholic fatty liver disease is most similar to those with no history of depression, rather than those with recent depressive symptoms in the last 12 months. Ellipses show the 70% confidence interval for each patient group according to the key. RD, recent depression (<12 months); ND, no history of depression; LD, lifetime history of depression (>12 months ago).
(TIF)

S4 Fig Orthogonal partial least squares discriminant analysis (OPLS-DA) of the serum metabolome and clinical chemistry does not identify a signature of lifetime depression. **A)** scores plot and **B)** mean accuracy of serum metabolic and clinical chemistry data, showing modest discrimination between those with any history of depression (recent or lifetime, N = 111) compared to those without (N = 107). Kolmogorov-Smirnov test, p<0.0001, accuracy 55.9% [95%CI 55.4–56.4].
(TIF)

S5 Fig Identification of recent depressive symptoms in this non-alcoholic fatty liver disease (NAFLD) cohort is robust to age differences. **A)** The original orthogonal partial least squares discriminant analysis (OPLS-DA) scores plot coloured by age. N = 81 NAFLD patients with recent depression, N = 137 patients with no/lifetime depression. **B)** The OPLS-DA scores plot of a randomly selected, age-matched subset taken from the original NAFLD cohort showing similar accuracy, specificity, and slightly reduced sensitivity after age-matching recent depression (N = 70) with non-recent depression (N = 70). **C)** Receiver operating characteristic (ROC) curve showing AUC ± 95% confidence intervals, based on the scores shown in B).
(TIF)

S6 Fig Orthogonal partial least squares discriminant analysis (OPLS-DA) scores plot showing no sex-specific clustering, with or between groups, in this cohort. N = 81 NAFLD patients with recent depression, N = 137 patients with no/lifetime depression. Ellipses show the 70% confidence interval for females and males in the scores plot.
(TIF)

S7 Fig Orthogonal partial least squares discriminant analysis (OPLS-DA) scores plot showing no clustering of patients on or off antidepressant treatment. N = 81 NAFLD patients with recent depression, N = 137 patients with no/lifetime depression. Ellipses show the 70% confidence interval for patients on and off antidepressants in the scores plot.
(TIF)

S1 Data (ZIP)
