# Supplementary material for: Depressive symptoms in non-alcoholic fatty liver disease are identified by perturbed lipid and lipoprotein metabolism
Source: PLoS One. 2022 Jan 6;17(1):e0261555. doi: 10.1371/journal.pone.0261555 (PMC8735618; doi:10.1371/journal.pone.0261555)
Supplement: S1 Appendix — (DOC) [file pone.0261555.s001.doc]

| 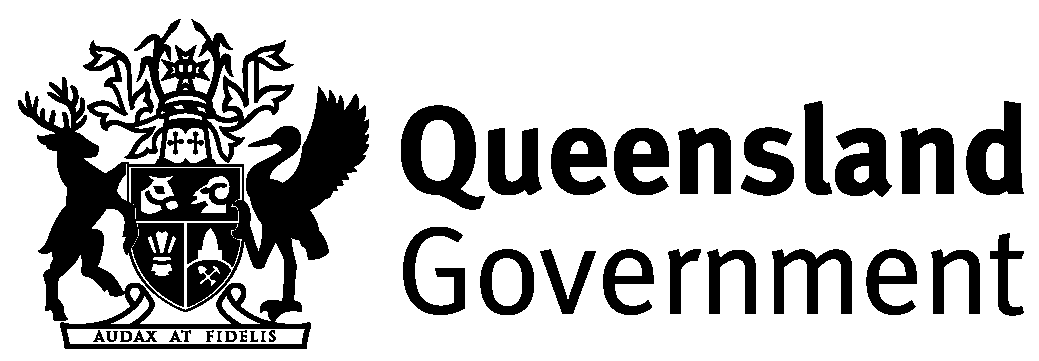  Princess Alexandra Hospital  NAFLD Assessment  Visit 1 | (Affix identification label here)  URN:  Family name:  Given name(s):  Address:  Date of birth: Sex:  M  F |
| --- | --- |
| | **NAFLD Study ID** |  | | | | | | | | | --- | --- | --- | --- | --- | --- | --- | --- | --- | | **Date Visit 1** | ____/ ____/ ________ | | | | | | | | |  | | | | | | | | | **General History** | Referred:  IPC  PAH Diabetes  Other: | | | | | | | | | Previous Scores: NAFLD Fibrosis Score:_______________ Fib 4:_______________ | | | | | | | | | Risk Group:  Low  Indeterminate  High | | | | | | | | | Date of NAFLD Diagnosis: ____/ ____/ ________ | | | | | | | | |  | | | | | | | | |  | | | | | | | | | Allergies: | | | | | | | | |  | | | | | | | | |  | | | | | | | | | **Social** | Country of Origin: | | | | | | | | | Race: | | | | | | | | |  | Caucasian Interpreter Required Yes  No | | | | | | | |  | African Language: | | | | | | | |  | Asian | | | | | | | |  | ATSI | | | | | | | |  | Pacific Islander | | | | | | | |  | Other: | | | | | | | | Partner: | | | | | | | | | Dependants: | | | | | | | | | Social Supports (describe): | | | | | | | | | Living Arrangements:  Alone  Partner  Share accommodation  with Dependents  Unknown | | | | | | | | |  | | | | | | | | | Pets: | | | | | | | | | Hobbies/interests: | | | | | | | | | Occupation: | | | | | | | | | Hours per week: Centrelink benefits: | | | | | | | | | No. of full years completed at school: | | | | | | | | | Tertiary study:  Nil  Certificate  Diploma  Degree  Post Graduate Qualification | | | | | | | | |  | | | | | | | | |  | | | | | | | | | **Vitals** | BP: Height: | | | | | | | | | Pulse: Weight: | | | | | | | | | Respirations: BMI: | | | | | | | | | Temperature: Girth: | | | | | | | | |  | | | | | | | | | **Weight History** | Heaviest Weight: Weight in 2010: | | | | | | | | | Lightest Adult Weight: Weight in 2000: | | | | | | | | |  | | | | | | | | | **Metabolic Risks** | Obesity  T2 Diabetes  HTN  Dyslipidaemia | | | | | | | | |  | | | | | | | | | **Treatment for NAFLD so far:** | Nil  Dietician (Community)  Dietician (Hospital)  Medication ____________________________  Complementary Practitioner  OTC / Herbal  Other: | | | | | | | | |  | | | | | | | | | **Med. Diagnoses** | IHD  Thyroid Disease  COPD  Psoriasis  CKD  Asthma  OSA  Malignancy | | | | | | | | | Other: | | | | | | | | |  | | | | | | | | |  | | | | | | | | | **Drug & Alcohol** | **Current (last 3 mths):** | | | **Past:** | | | | | | Alcohol | | | Alcohol | | | | | | Tobacco ____cigs /day x _____yrs | | | Tobacco ____cigs /day x _____yrs | | | | | | Recreational Drugs | | | Recreational Drugs | | | | | | Analgesia | | | Analgesia | | | | | | Other: | | | Other: | | | | | | Audit Questionnaire Completed: Yes  No | | | | | | | | | Audit Questionnaire Result: | | | | | | | | |  | | | | | | | | | **Mental Health** | Depression - on medication Yes  No | | | | | | | | | Anxiety - on medication Yes  No | | | | | | | | | Other: | | | | | | | | | Problems in last 12 months? Yes  No | | | | | | | | | Admitted to Hospital? Yes  No | | | | | | | | | Previous / current major stressors: | | | | | | | | |  | | | | | | | | | Previous / current coping strategies: | | | | | | | | |  | | | | | | | | | Effectiveness of strategies: | | | | | | | | |  | | | | | | | | | Psychiatrist: Yes  No  Name: | | | | | | | | | Psychologist / Counsellor: Yes  No  Name: | | | | | | | | |  | | | | | | | | | **Medications**  **(Including OTC / Herbal)** | **Medication Name** | | **Dose** | | **Route** | **Frequency** | **Start Date** | **End Date** | |  | |  | |  |  |  |  | |  | |  | |  |  |  |  | |  | |  | |  |  |  |  | |  | |  | |  |  |  |  | |  | |  | |  |  |  |  | |  | |  | |  |  |  |  | |  | |  | |  |  |  |  | |  | |  | |  |  |  |  | |  | |  | |  |  |  |  | |  | |  | |  |  |  |  | |  | |  | |  |  |  |  | | **Imaging**  **(most recent)** | Abdominal Ultrasound – Date: ____/ ____/ ________ Where? | | | | | | | | | Abdominal CT Scan – Date: ____/ ____/ ________ Where? | | | | | | | | | Abdominal MRI – Date: ____/ ____/ ________ Where? | | | | | | | | | Other: | | | | | | | | |  | | | | | | | | |  | | | | | | | | | **Imaging Result** | Hepatomegaly (size): | | | | | | | | | Splenomegaly (size): | | | | | | | | | Varices | | | | | | | | | Nodular / irregular liver surface | | | | | | | | | Description: | | | | | | | | |  | | | | | | | | |  | | | | | | | | |  | | | | | | | | | **Physical Examination** | Digestive / Liver: | | | | | | | | | Cardiovascular: | | | | | | | | | Respiratory: | | | | | | | | | Endocrine: | | | | | | | | | Muscular: | | | | | | | | | Skeletal: | | | | | | | | | Lymph nodes: | | | | | | | | | Nervous system: | | | | | | | | | Malignancy (Type and Date): | | | | | | | | |  | | | | | | | | |  | | | | | | | | |  | | | | | | | |   **INSERT FORM TITLE** | |
|  | |

| 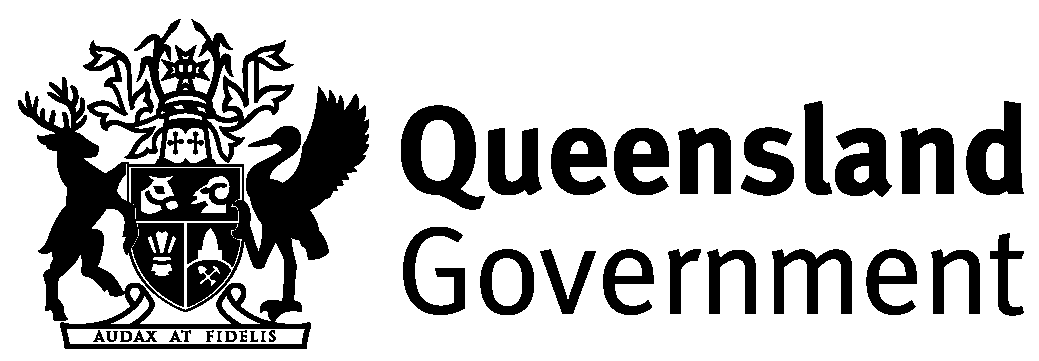  Princess Alexandra Hospital  NAFLD Assessment  Visit 1 | (Affix identification label here)  URN:  Family name:  Given name(s):  Address:  Date of birth: Sex:  M  F |
| --- | --- |
| | Screening Bloods  (Fasting) | Creat. |  | Free T4 |  | HIV |  | | --- | --- | --- | --- | --- | --- | --- | | eGFR |  | TSH |  | HBsAg |  | | Protein |  | Ceruloplasmin |  | HBsAb |  | | Albumin |  | AFP |  | HBcAb |  | | Bilirubin |  | ά1AT |  | HCV Ab |  | | Bili (conj) |  | ά1AT Genotype |  | HCV RNA |  | | ALP |  | Transferrin Sat. |  | ANA |  | | GGT |  | Ferritin |  | SMA |  | | ALT |  | Hb |  | AMA |  | | AST |  | Plt |  | Cholesterol |  | | CK |  | WBC |  | Triglyceride |  | | Glucose |  | MCV |  | HDL |  | | HBA1C |  | INR |  | LDL |  | | C-Peptide |  | PT |  |  | | | Insulin |  |  | |  | | | Research Bloods: Yes  No  Serum Tubes ______ EDTA Tubes ______ | | | | | | | Research Bloods collected by:  Path QLD  Study staff | | | | | | |  | | | | | | | Post Visit 1 Referrals | Ultrasound: Yes  No | | | | | | | Fibroscan: Yes  No | | | | | | | Dietician: Yes  No | | | | | | | Psych: Yes  No | | | | | | | ECG: Yes  No | | | | | | |  | | | | | | |  | Patient Education attended: Yes  No | | | | | | |  | Fatty Liver Disease Flyer Provided: Yes  No | | | | | | |  | Follow up appointment made: | | | | | | |  | Letter to GP: Yes  No | | | | | | |  | Letter to Endocrine: Yes  No | | | | | | | |
|  | |
