# Supplementary material for: Depressive symptoms in non-alcoholic fatty liver disease are identified by perturbed lipid and lipoprotein metabolism
Source: PLoS One. 2022 Jan 6;17(1):e0261555. doi: 10.1371/journal.pone.0261555 (PMC8735618; doi:10.1371/journal.pone.0261555)
Supplement: S1 Table — (DOCX) [file pone.0261555.s002.docx]

| **Variable** | **Mean VIP score** |
| --- | --- |
| Triglycerides | 2.67 |
| HDL | 2.26 |
| eGFR | 1.35 |
| HBA1c | 1.14 |
| LDL | 1.06 |
| Cholesterol | 0.90 |
| BMI | 0.56 |
| Ferritin | 0.11 |
