# Supplementary material for: Depressive symptoms in non-alcoholic fatty liver disease are identified by perturbed lipid and lipoprotein metabolism
Source: PLoS One. 2022 Jan 6;17(1):e0261555. doi: 10.1371/journal.pone.0261555 (PMC8735618; doi:10.1371/journal.pone.0261555)
Supplement: S2 Table — (DOCX) [file pone.0261555.s003.docx]

|  | Depressive symptoms | |  |
| --- | --- | --- | --- |
|  | Recent depressive symptoms (<12months) N=81 | No depression or no symptoms for >12months N=137 | ROC AUC Value (95% CI) |
| Serum Triglycerides (mmol/L)* | 2.0 (1.6-2.9) | 1.7 (1.2-2.0) | 0.64 (0.57-0.71) |
| Serum HDL (mmol/L)* | 1.0 (0.8-1.1) | 1.2 (0.9-1.3) | 0.61 (0.53-0.68) |
| HBA1c (%)* | 7.7 (6.6-9.0) | 7.2 (6.0-8.6) | 0.58 (0.50-0.65) |
| eGFR (mL/min/1.73m²)* | 90 (80-90) | 88 (69-90) | 0.56 (0.49-0.63) |
| Serum Cholesterol (mmol/L)* | 4.5 (3.6-5.5) | 4.6 (3.5-5.8) | 0.55 (0.47-0.63) |
| Serum LDL (mmol/L)* | 2.6 (1.7-3.2) | 2.5 (1.8-3.8) | 0.54 (0.46-0.62) |
| Serum Ferritin (µg/L)* | 70 (38-141) | 91 (48-159) | 0.54 (0.46-0.62) |
| Serum ALT (IU/mL)* | 31 (22-51) | 28 (20-45) | 0.54 (0.45-0.61) |
| LSM kPa*^#^ | 5.6 (4.8-7.9) | 5.8 (4.6-8.1) | 0.51 (0.44-0.59) |
| Serum AST (IU/mL)* | 21 (15-28) | 21 (15-32) | 0.48 (0.44-0.59) |

*Continuous data (median [IQR]) analysed using a receiver operator characteristic (ROC) curve, reporting the area under the curve (AUC). ^#^LSM (liver stiffness measurement) presented for 197/218 patients with a reliable measurement. eGFR, estimated glomerular filtration rate; ALT, alanine aminotransferase; AST, aspartate aminotransferase; HBA1c, glycated haemoglobin; HDL, high-density lipoprotein; LDL, low-density lipoprotein.
