# Supplementary material for: Depressive symptoms in non-alcoholic fatty liver disease are identified by perturbed lipid and lipoprotein metabolism
Source: PLoS One. 2022 Jan 6;17(1):e0261555. doi: 10.1371/journal.pone.0261555 (PMC8735618; doi:10.1371/journal.pone.0261555)
Supplement: S4 Table — (DOCX) [file pone.0261555.s005.docx]

| **Metabolite [ppm] or clinical variable [clin]** | **Mean VIP score** | **Metabolite [ppm] or clinical variable [clin]** | **Mean VIP score** |
| --- | --- | --- | --- |
| Glutamine [2.44-49] | 1.73 | X.1.04....1.05. | 0.92 |
| Triglyceride [1.34-43] | 1.58 | X.3.47....3.47..1 | 0.92 |
| Lactate/TG [1.32-34] | 1.55 | X.3.83....3.84. | 0.92 |
| TG/VLDL [1.22-32] | 1.53 | X.3.47....3.47. | 0.92 |
| Beta-CH2 lipid [1.55-64] | 1.52 | X.3.74....3.74. | 0.92 |
| VLDL/TG [0.87-92] | 1.52 | X.3.85....3.85. | 0.92 |
| Alpha-CH2 lipid [2.23-28] | 1.50 | X.0.94....0.95. | 0.92 |
| X.3.15....3.15. | 1.49 | X.3.46....3.47. | 0.91 |
| X.3.30....3.30. | 1.49 | X.3.45....3.46. | 0.91 |
| X.2.41....2.41. | 1.48 | X.3.73....3.73. | 0.91 |
| Triglycerides [clin] | 1.47 | X.3.40....3.41. | 0.91 |
| X.5.19....5.23. | 1.45 | X.3.42....3.42. | 0.91 |
| X.3.68....3.70. | 1.38 | X.3.84....3.84. | 0.91 |
| X.7.36....7.38. | 1.33 | X.4.13....4.15. | 0.91 |
| X.2.28....2.28. | 1.32 | X.5.18....5.19. | 0.91 |
| X.3.28....3.28. | 1.29 | X.3.47....3.47..2 | 0.90 |
| X.3.29....3.30. | 1.28 | X.3.75....3.76. | 0.90 |
| X.4.17....4.17. | 1.28 | X.3.46....3.46. | 0.90 |
| X.1.19....1.21. | 1.24 | X.3.85....3.86. | 0.90 |
| X.2.82....2.83. | 1.24 | X.3.89....3.91. | 0.89 |
| eGFR [clin] | 1.22 | X.2.30....2.30. | 0.89 |
| X.4.15....4.16. | 1.20 | X.3.40....3.40. | 0.89 |
| X.2.39....2.43. | 1.18 | X.3.54....3.55. | 0.89 |
| X.2.78....2.82. | 1.11 | X.3.65....3.68. | 0.88 |
| X.3.27....3.27. | 1.07 | X.3.43....3.43. | 0.88 |
| X.7.14....7.16. | 1.05 | Cholesterol [clin] | 0.87 |
| GlycA [2.04-05] | 1.05 | X.3.48....3.48. | 0.87 |
| X.5.23....5.25. | 1.04 | X.3.78....3.78. | 0.87 |
| X.2.29....2.30. | 1.03 | X.3.72....3.72..1 | 0.86 |
| BMI [clin] | 1.02 | X.1.92....1.92. | 0.86 |
| X.2.34....2.36. | 1.02 | X.3.82....3.83. | 0.84 |
| X.0.98....1.00. | 1.01 | X.3.76....3.77. | 0.84 |
| X.3.11....3.11. | 0.99 | X.3.77....3.78. | 0.84 |
| X.3.36....3.36. | 0.99 | X.3.39....3.39. | 0.83 |
| X.2.83....2.86. | 0.97 | X.3.56....3.56. | 0.83 |
| X.2.05....2.10. | 0.97 | X.1.01....1.02. | 0.83 |
| X.3.24....3.25. | 0.95 | X.3.04....3.05. | 0.79 |
| X.3.26....3.26. | 0.94 | X.3.49....3.50. | 0.78 |
| X.3.41....3.42. | 0.93 | X.1.93....1.95. | 0.78 |
| X.3.84....3.85. | 0.93 | X.3.23....3.24. | 0.77 |
| X.3.53....3.54. | 0.93 | X.2.31....2.31. | 0.76 |
| X.2.16....2.17. | 0.76 | X.7.19....7.20. | 0.14 |
| X.3.71....3.72. | 0.75 | X.2.93....2.93. | 0.14 |
| X.4.10....4.13. | 0.75 |  |  |
| X.3.70....3.71. | 0.74 |  |  |
| X.3.50....3.51. | 0.74 |  |  |
| X.3.72....3.72. | 0.72 |  |  |
| X.2.41....2.42. | 0.72 |  |  |
| X.1.47....1.50. | 0.67 |  |  |
| X.3.96....3.96. | 0.67 |  |  |
| X.2.42....2.43. | 0.67 |  |  |
| HDL [clin] | 0.67 |  |  |
| X.3.93....3.94. | 0.67 |  |  |
| X.3.01....3.02. | 0.62 |  |  |
| X.6.89....6.91. | 0.22 |  |  |
| X.2.39....2.40. | 0.62 |  |  |
| X.3.79....3.79. | 0.61 |  |  |
| X.3.04....3.04. | 0.58 |  |  |
| X.3.95....3.95. | 0.58 |  |  |
| X.2.40....2.41. | 0.58 |  |  |
| LDL [clin] | 0.54 |  |  |
| HBA1c [clin] | 0.53 |  |  |
| X.3.97....4.00. | 0.52 |  |  |
| Fibroscan [clin] | 0.49 |  |  |
| X.3.06....3.07. | 0.49 |  |  |
| X.3.02....3.03. | 0.47 |  |  |
| X.3.61....3.62. | 0.44 |  |  |
| X.1.90....1.91. | 0.43 |  |  |
| X.3.05....3.06. | 0.43 |  |  |
| X.1.89....1.90. | 0.42 |  |  |
| X.2.32....2.32. | 0.41 |  |  |
| X.2.37....2.37. | 0.41 |  |  |
| X.3.19....3.23. | 0.41 |  |  |
| X.2.52....2.53. | 0.40 |  |  |
| X.3.57....3.59. | 0.40 |  |  |
| HDL [0.81-87] | 0.35 |  |  |
| X.1.68....1.76. | 0.35 |  |  |
| X.3.26....3.27. | 0.33 |  |  |
| X.4.05....4.06. | 0.33 |  |  |
| X.3.86....3.87. | 0.31 |  |  |
| X.2.54....2.55. | 0.30 |  |  |
| X.2.12....2.16. | 0.30 |  |  |
| X.2.68....2.69. | 0.30 |  |  |
| X.2.66....2.66. | 0.27 |  |  |
| X.2.71....2.71. | 0.27 |  |  |
| X.2.64....2.65. | 0.21 |  |  |
| Ferritin [clin] | 0.19 |  |  |
