# Supplementary material for: Depressive symptoms in non-alcoholic fatty liver disease are identified by perturbed lipid and lipoprotein metabolism
Source: PLoS One. 2022 Jan 6;17(1):e0261555. doi: 10.1371/journal.pone.0261555 (PMC8735618; doi:10.1371/journal.pone.0261555)
Supplement: S6 Table — (DOCX) [file pone.0261555.s007.docx]

|  | Clinical marker | |
| --- | --- | --- |
| Serum metabolite [chemical shift ppm] | ELF Score | LSM (kPa) |
| HDL [0.81-0.87] | 0.12 (0.23) | -0.031 (0.69) |
| VLDL/TG [0.87-0.92] | 0.052 (0.58) | 0.17 (0.10) |
| TG/VLDL [1.22-1.32] | 0.068 (0.50) | 0.17 (0.10) |
| TG [1.34-1.43] | 0.050 (0.58) | 0.15 (0.16) |
| GlycA [2.04-2.05] | 0.045 (0.59) | 0.11 (0.23) |
| Glutamine [2.43-2.48] | 0.12 (0.23) | -0.11 (0.25) |

Spearman rank correlation co-efficient (FDR-adjusted p-value). Method: Two-stage linear step-up procedure of Benjamini, Krieger and Yekutieli.
