# Supplementary figures and images for: Depressive symptoms in non-alcoholic fatty liver disease are identified by perturbed lipid and lipoprotein metabolism
Source: PLoS One. 2022 Jan 6;17(1):e0261555. doi: 10.1371/journal.pone.0261555 (PMC8735618; doi:10.1371/journal.pone.0261555)

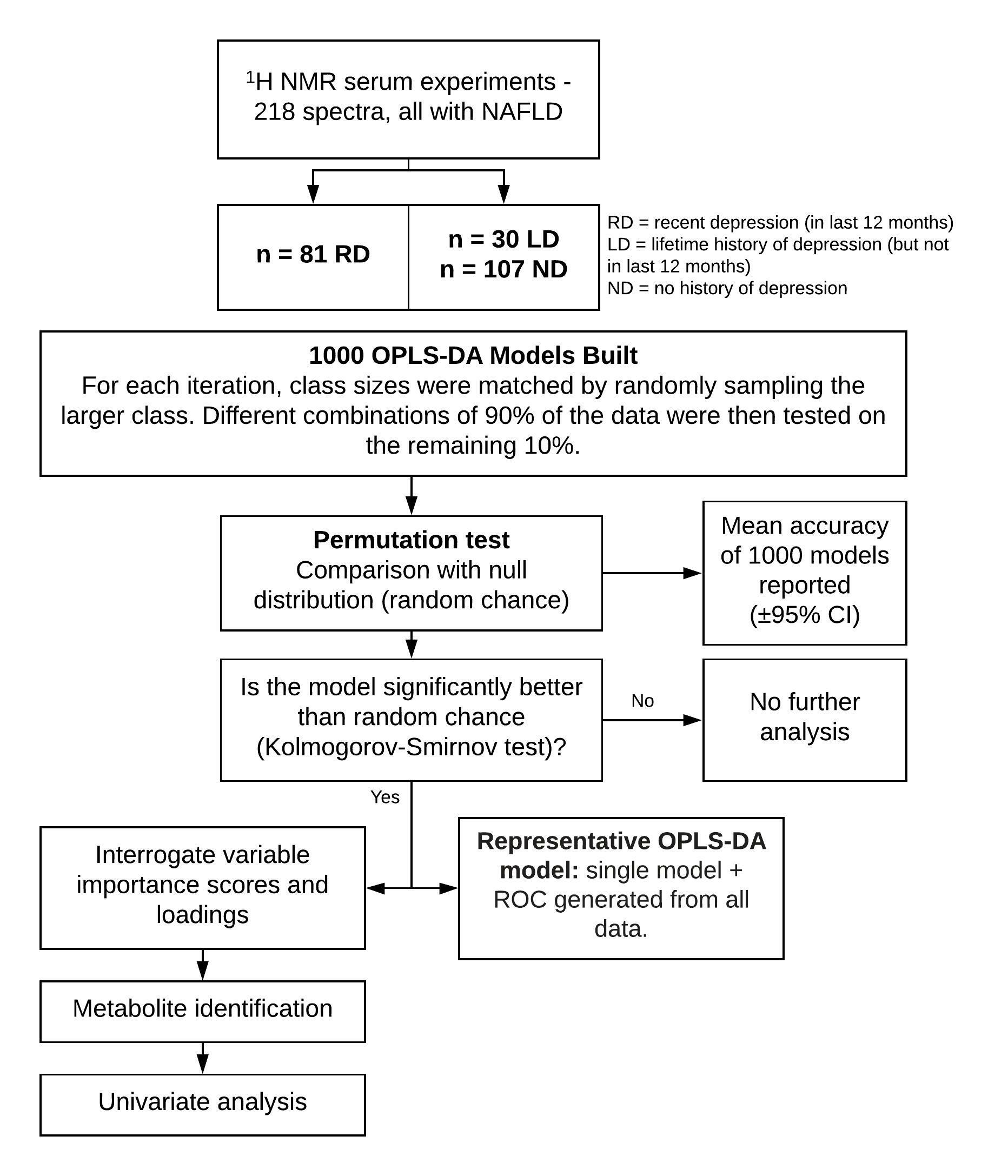

Supplement: S1 Fig — (TIF) [file pone.0261555.s008.tif]

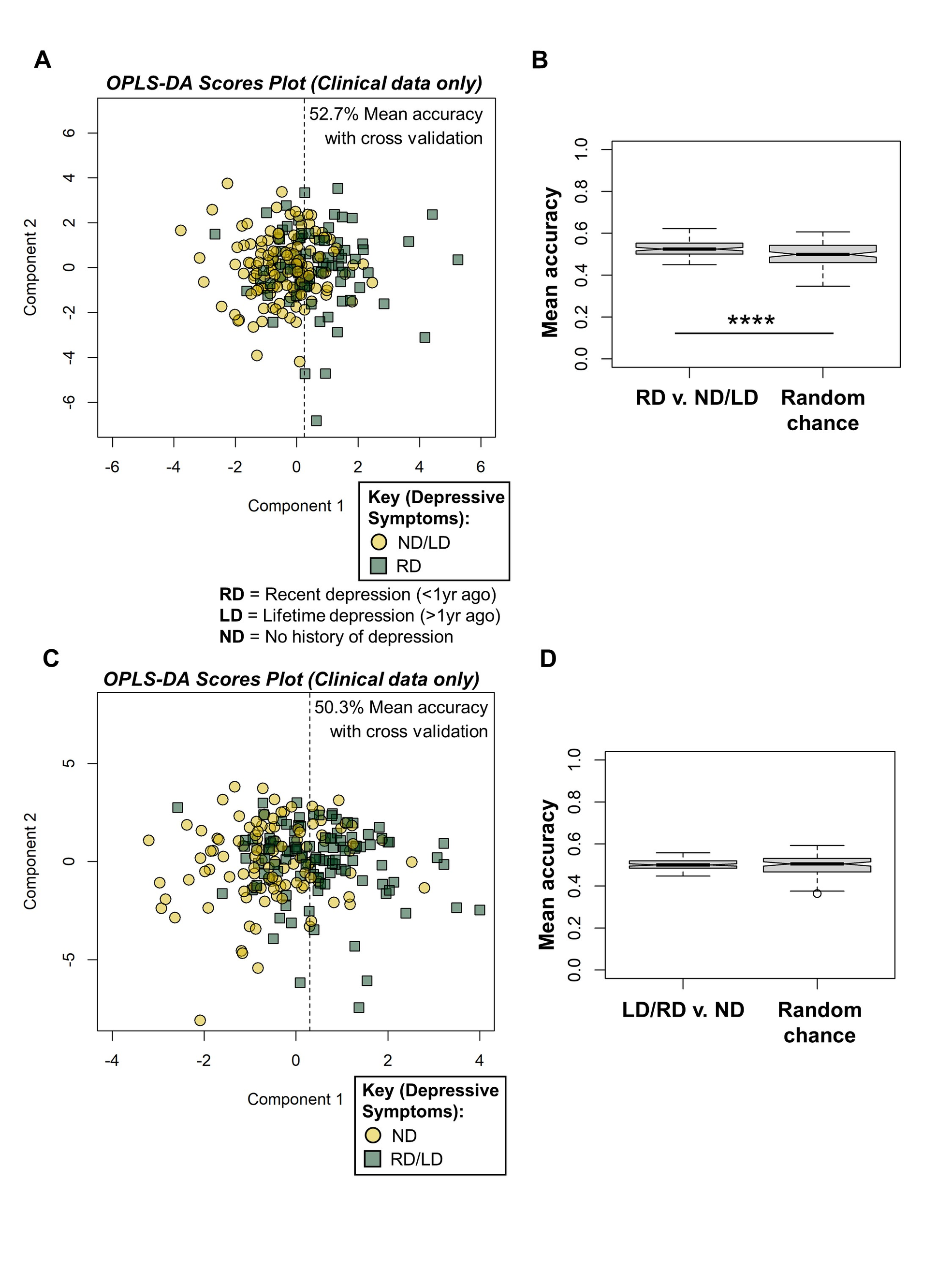

Supplement: S2 Fig — A) OPLS-DA scores plot comparing recent depressive symptoms (in the last 12 months, N = 81) against no recent depressive symptoms in people with or without lifetime depression (N = 137). B) Corresponding mean accuracy of OPLS-DA models compared to null distribution, 52.7% [95%CI 52.0–53.4], Kolmogorov-Smirnov test p<0.0001. C) OPLS-DA scores plot comparing lifetime/recent depression (N = 111) against NAFLD patients with no history of depression (N = 107). D) Corresponding mean accuracy of OPLS-DA models compared to null distribution, 50.3% [95%CI 49.8–50.7], Kolmogorov-Smirnov test p = 0.20. (TIF) [file pone.0261555.s009.tif]

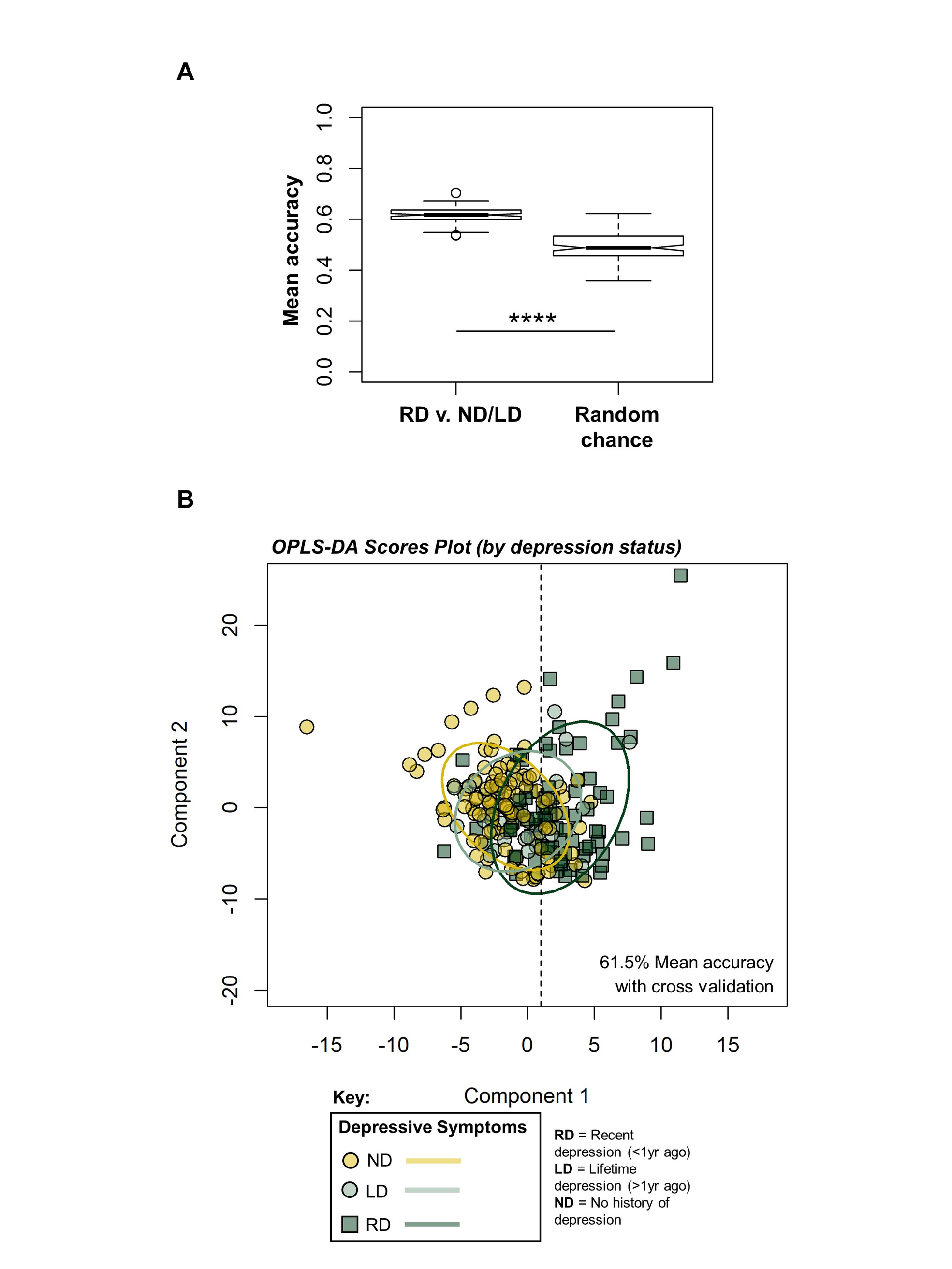

Supplement: S3 Fig — A) Mean accuracy of OPLS-DA models (61.5% [CI 60.9–62.1]). 10-fold cross validation and permutation testing with 100 repetitions verified that the model was significantly better than random chance (Kolmogorov-Smirnov test, p < 0.0001). N = 81 NAFLD patients with recent depression, N = 137 patients with no/lifetime depression. B) OPLS-DA scores plot coloured by recent (N = 81), lifetime (N = 30), and no history of depression (N = 107) illustrates that the serum metabolic and clinical profile of lifetime depression in non-alcoholic fatty liver disease is most similar to those with no history of depression, rather than those with recent depressive symptoms in the last 12 months. Ellipses show the 70% confidence interval for each patient group according to the key. RD, recent depression (<12 months); ND, no history of depression; LD, lifetime history of depression (>12 months ago). (TIF) [file pone.0261555.s010.tif]

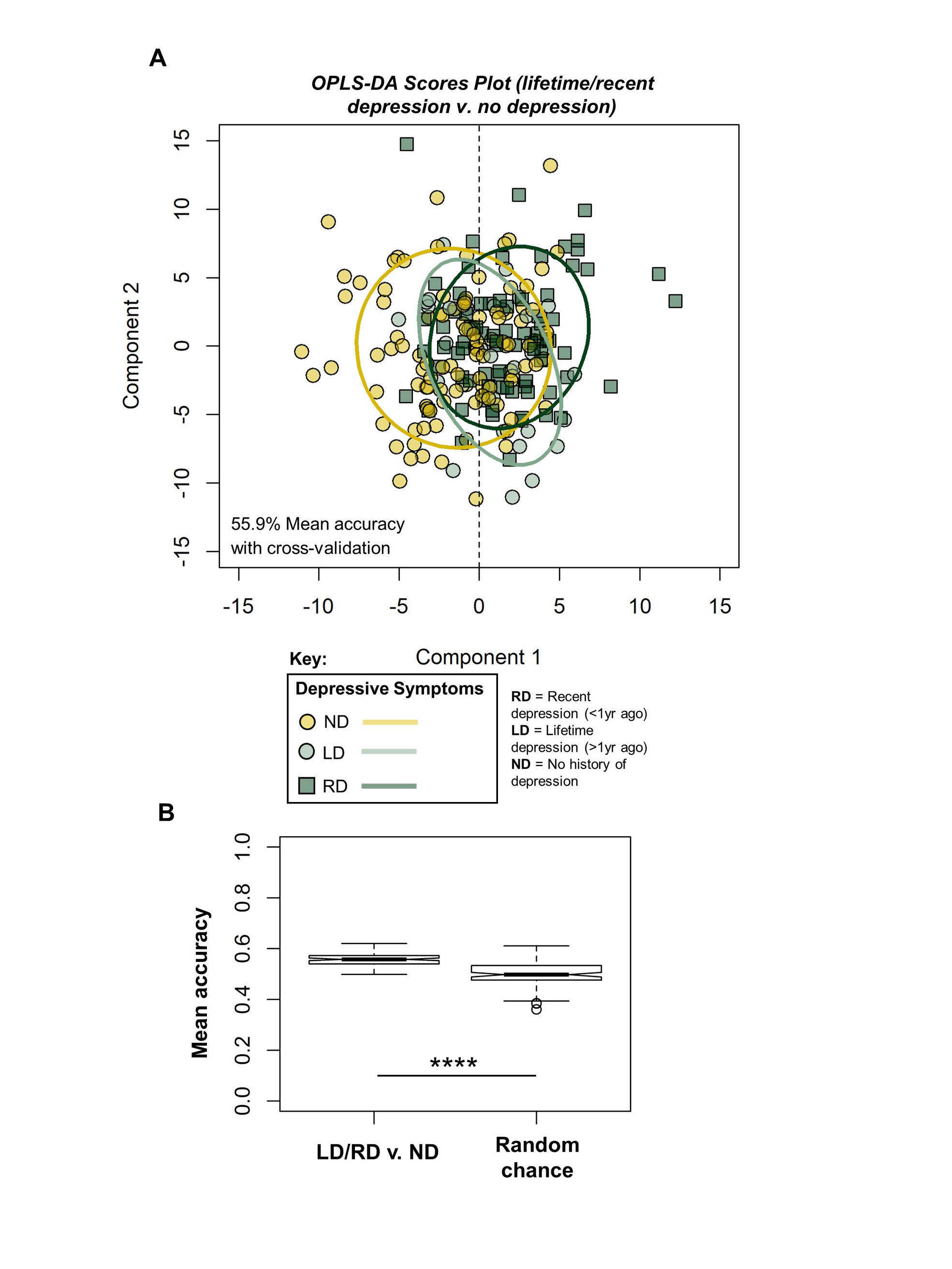

Supplement: S4 Fig — A) scores plot and B) mean accuracy of serum metabolic and clinical chemistry data, showing modest discrimination between those with any history of depression (recent or lifetime, N = 111) compared to those without (N = 107). Kolmogorov-Smirnov test, p<0.0001, accuracy 55.9% [95%CI 55.4–56.4]. (TIF) [file pone.0261555.s011.tif]

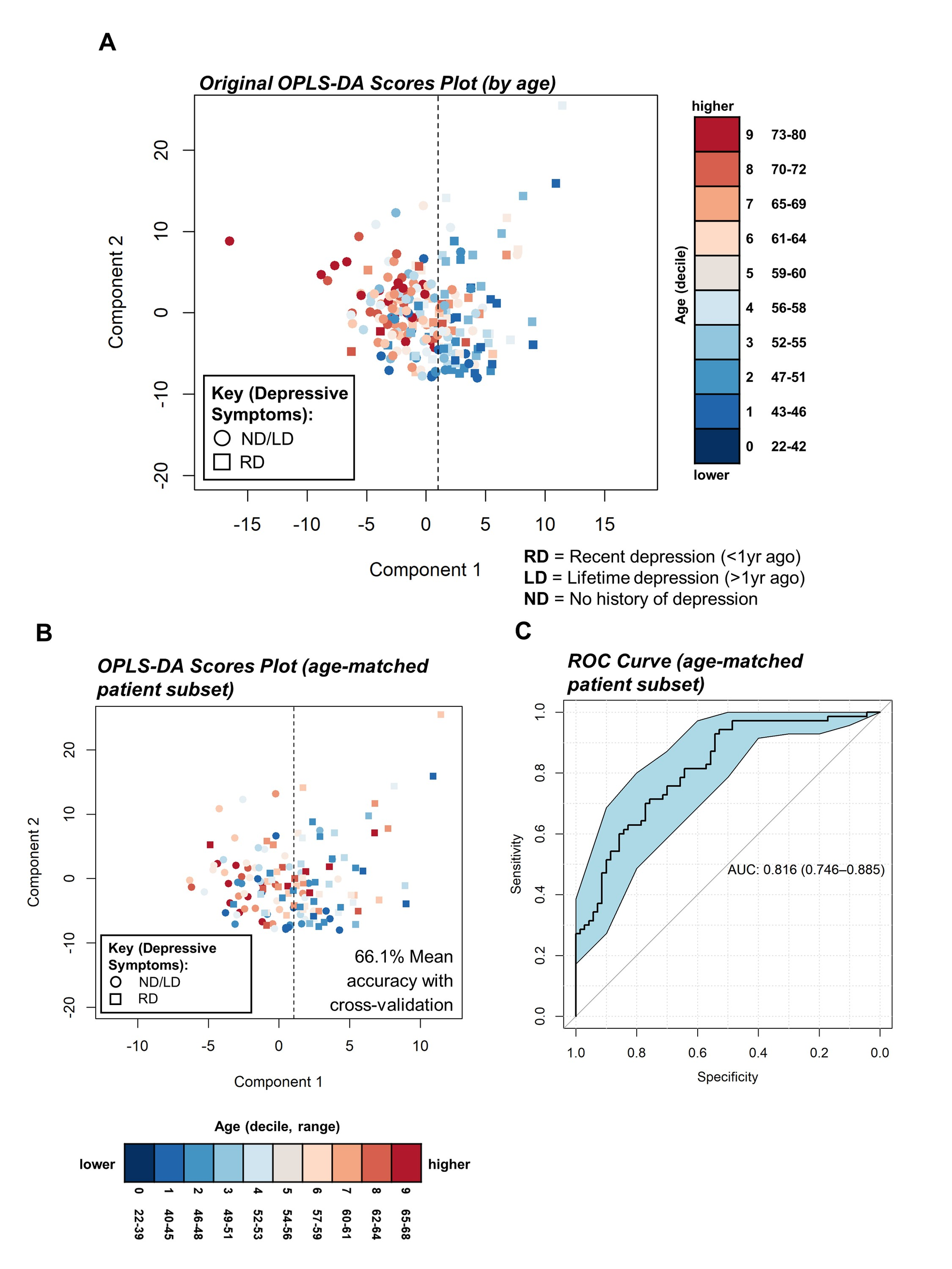

Supplement: S5 Fig — A) The original orthogonal partial least squares discriminant analysis (OPLS-DA) scores plot coloured by age. N = 81 NAFLD patients with recent depression, N = 137 patients with no/lifetime depression. B) The OPLS-DA scores plot of a randomly selected, age-matched subset taken from the original NAFLD cohort showing similar accuracy, specificity, and slightly reduced sensitivity after age-matching recent depression (N = 70) with non-recent depression (N = 70). C) Receiver operating characteristic (ROC) curve showing AUC ± 95% confidence intervals, based on the scores shown in B). (TIF) [file pone.0261555.s012.tif]

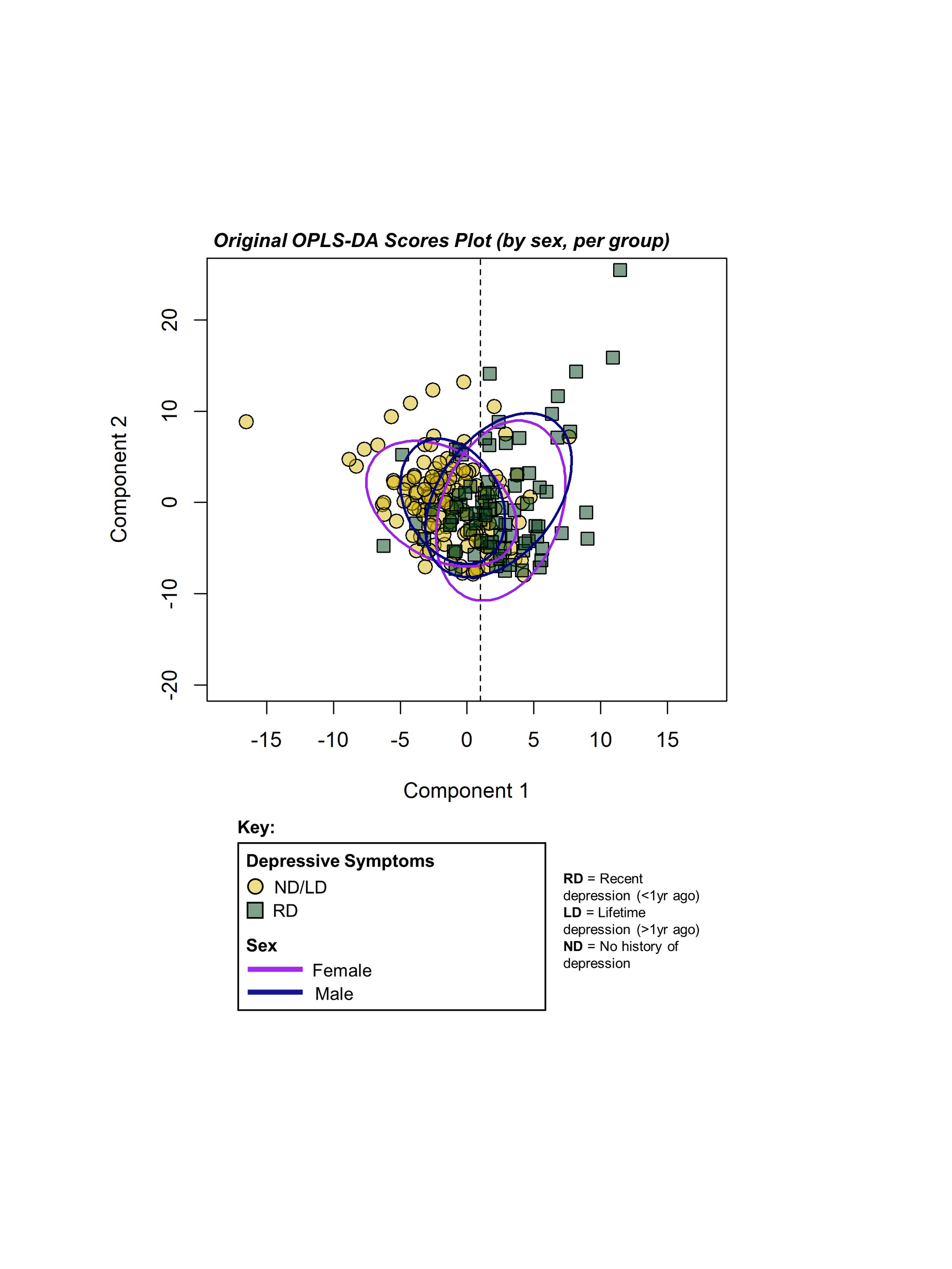

Supplement: S6 Fig — N = 81 NAFLD patients with recent depression, N = 137 patients with no/lifetime depression. Ellipses show the 70% confidence interval for females and males in the scores plot. (TIF) [file pone.0261555.s013.tif]

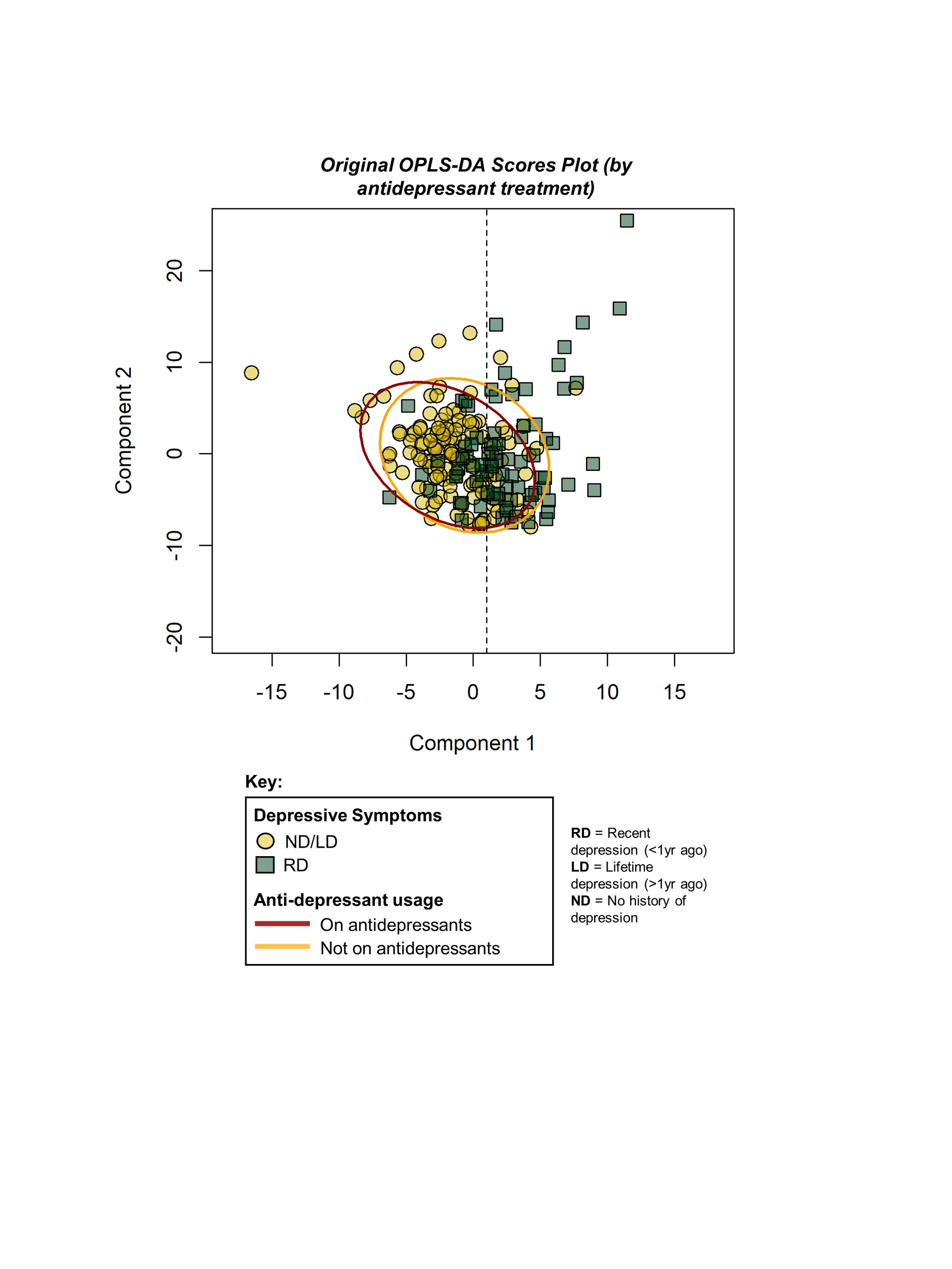

Supplement: S7 Fig — N = 81 NAFLD patients with recent depression, N = 137 patients with no/lifetime depression. Ellipses show the 70% confidence interval for patients on and off antidepressants in the scores plot. (TIF) [file pone.0261555.s014.tif]
